# Amine-Functionalized ZnO Nanosheets for Efficient CO_2_ Capture and Photoreduction

**DOI:** 10.3390/molecules201018847

**Published:** 2015-10-16

**Authors:** Yusen Liao, Zhaoning Hu, Quan Gu, Can Xue

**Affiliations:** School of Materials Science and Engineering, Nanyang Technological University, 639798 Singapore, Singapore; E-Mails: liao0045@e.ntu.edu.sg (Y.L.); huzh0007@e.ntu.edu.sg (Z.H.); guquan@ntu.edu.sg (Q.G.)

**Keywords:** photocatalysis, CO_2_ reduction, solar fuels, zinc oxide

## Abstract

Amine-functionalized ZnO nanosheets were prepared through a one-step hydrothermal method by using monoethanolamine, which has a hydroxyl group, for covalent attachment on ZnO and a primary amine group to supply the amine-functionalization. We demonstrate that the terminal amine groups on ZnO surfaces substantially increase the capability of CO_2_ capture via chemisorption, resulting in effective CO_2_ activation. As a result, the photogenerated electrons from excited ZnO can more readily reduce the surface-activated CO_2_, which thereby enhances the activity for photocatalytic CO_2_ reduction.

## 1. Introduction

Nowadays, the increasing level of CO_2_ concentration from the use of fossil fuels has become the most serious environmental concern related to global warming and climate change. Researchers have realized that photocatalytic conversion of CO_2_ into hydrocarbons by means of solar energy would be an ideal way to lower down the CO_2_ concentration in the atmosphere and generate sustainable chemical fuels in the meantime. A pioneering study on the reduction of CO_2_ was demonstrated by Inoue *et al.* in 1979 by using semiconductor photocatalysts [1]. Afterwards, many scientific studies have been devoted to the development of efficient photocatalysts for CO_2_ photoreduction [2,3,4,5,6,7,8,9,10,11,12,13,14]. Among them, ZnO-based nanostructures with different sizes and morphologies have been extensively reported as photocatalysts since they are stable, non-toxic, and low cost [15,16,17,18,19,20]. However, the efficiencies for CO_2_ photoreduction of these ZnO-based photocatalysts were usually unsatisfactory [21,22], which might be ascribed to the low affinity between CO_2_ and ZnO surfaces [23].

As is well known, the amine functionalization can effectively assist immobilization of CO_2_ and has been extensively used for CO_2_ capture in industry [24,25]. Tremendous efforts have been made to introduce amine groups onto solid material surfaces to promote CO_2_ capture [26,27]. The chemical interactions between amine groups and CO_2_ lead to the formation of carbamate (or bicarbamate) that can transform into carbonate upon hydrolysis [28]. In this work, we report a straightforward method to prepare amine-functionalized ZnO nanosheets by using monoethanolamine (MEA) that possesses a hydroxyl (-OH) group for covalent attachment on ZnO and a primary amine (-NH_2_) group to endow an amine-functionalized surface. We demonstrate that the terminal amine groups on ZnO surfaces substantially improve the CO_2_ adsorption, which consequently results in significantly enhanced photocatalytic activity for CO_2_ reduction as compared to the clean ZnO without surface amine groups.

## 2. Results and Discussion

**Figure 1:**
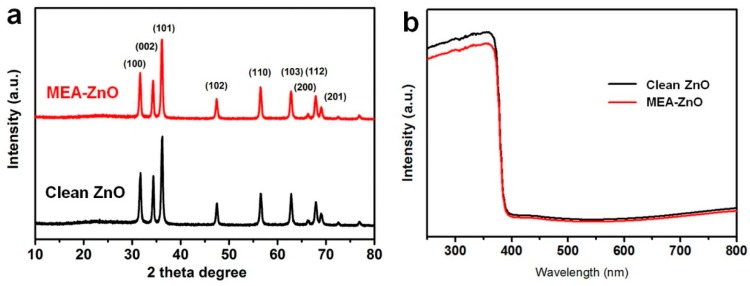
(**a**) XRD patterns of MEA–ZnO and clean ZnO. Both samples can be indexed as wurtzite ZnO; (**b**) UV-Vis spectra of MEA–ZnO and clean ZnO.

**Figure 2:**
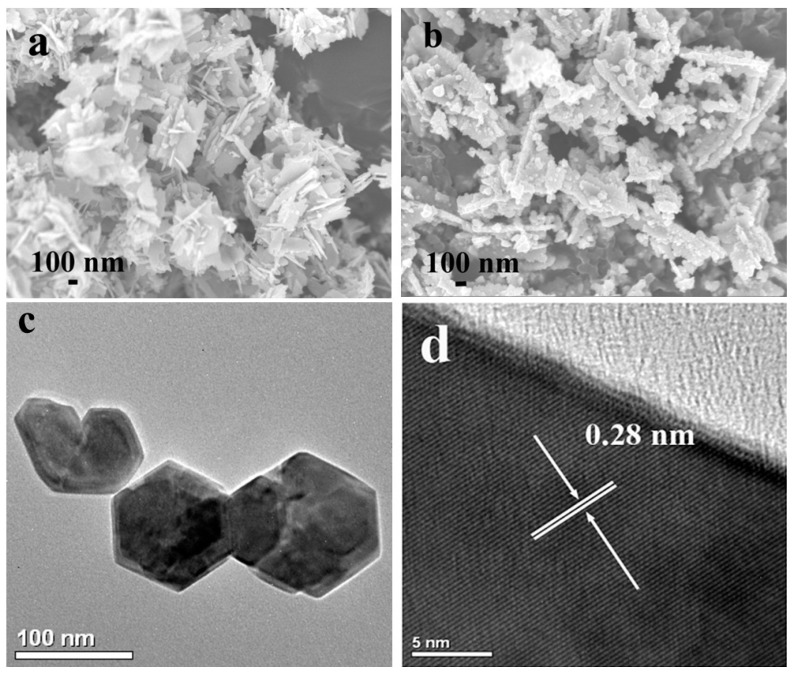
(**a**) SEM images of MEA–ZnO; (**b**) SEM image of clean ZnO; (**c**) TEM image of MEA–ZnO nanosheets; (**d**) HRTEM image of a representative MEA–ZnO nanosheet.

Figure 1a shows the X-ray diffraction (XRD) patterns of the prepared MEA–ZnO sample and clean ZnO sample. These two samples exhibit almost the same diffraction lines which can be indexed to wurtzite ZnO. This indicates that the involvement of MEA into the reaction did not alter the crystal structure of ZnO. The absorption spectra of both samples also appear to have almost no difference, as shown in Figure 1b. The SEM image (Figure 2a) of the MEA–ZnO sample confirms the nanosheet morphology, and the high-resolution TEM (HRTEM) image (Figure 2a inset) shows a lattice fringe of d = 0.28 nm, corresponding to the (100) interplanar spacing of wurtzite ZnO [29]. In comparison, the clean ZnO sample prepared without MEA appears as irregular particles, as shown in Figure 2b. The sheet-like growth of ZnO is probably due to attached MEA molecules that suppress the intrinsically anisotropic growth of ZnO along the (0001) direction. As such, the ZnO crystal growth would intend to proceed sideways towards a nanosheet [15].

**Figure 3:**
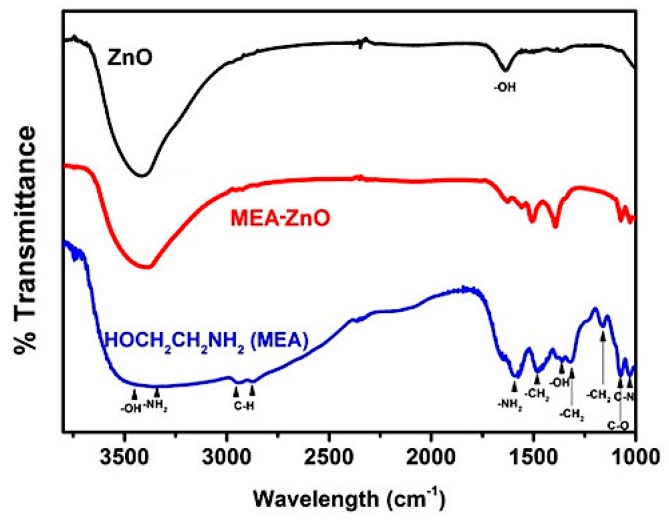
FT-IR spectra of clean ZnO, MEA–ZnO, and the pure MEA molecule.

For the MEA–ZnO sample, the nature of covalent attachment of MEA on the ZnO surface is verified by Fourier transform infrared (FT-IR) spectroscopy. Prior to the measurement, the as-prepared MEA–ZnO sample was annealed at 250 °C under Ar atmosphere to remove the physisorbed MEA since the boiling point of MEA is ~170 °C. The FT-IR spectrum of the MEA–ZnO sample after annealing (Figure 3) still shows most of the signature peaks of MEA. The bands at 2964, 2919, and 1399 cm^−1^ can be assigned to the C–H stretch of MEA. The peaks at 1073 and 1028 cm^−1^ are attributed to the C–O stretch and C–N stretch, respectively, and the band at 1560 cm^−1^ is owing to the N-H stretch of MEA on the ZnO surface [28,30]. These IR results indicate that MEA molecules are successfully covalently binding on ZnO surfaces.

**Figure 4:**
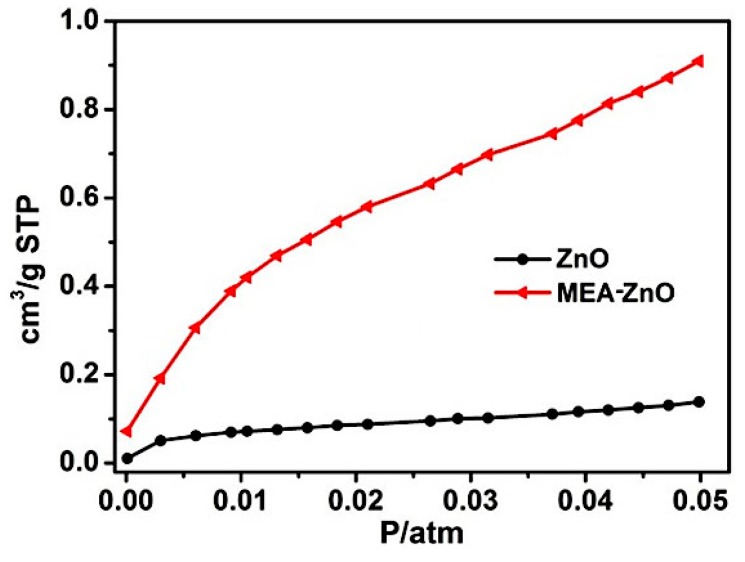
CO_2_ adsorption isotherms of MEA–ZnO and clean ZnO.

The effect of amine functionalization by MEA is examined through the CO_2_ adsorption test. As shown in Figure 4, the MEA–ZnO exhibits much higher (approximately six times) CO_2_ uptake capability (0.87 cm^3^·g^−1^) than the clean ZnO (0.13 cm^3^·g^−1^), while in comparison, the difference in their surface area is quite minor (15.1 m^2^·g^−1^ for MEA–ZnO *vs.* 9.6 m^2^·g^−1^ for clean ZnO). This result proves that the amine-functionalized ZnO surface can greatly enhance CO_2_ adsorption. Moreover, the MEA–ZnO shows a dramatic rise in CO_2_ uptake along with elevated CO_2_ pressure, while the clean ZnO shows almost linearly increased CO_2_ adsorption. This observation suggests that specific interactions between CO_2_ and MEA–ZnO occur during the CO_2_ adsorption process.

**Figure 5:**
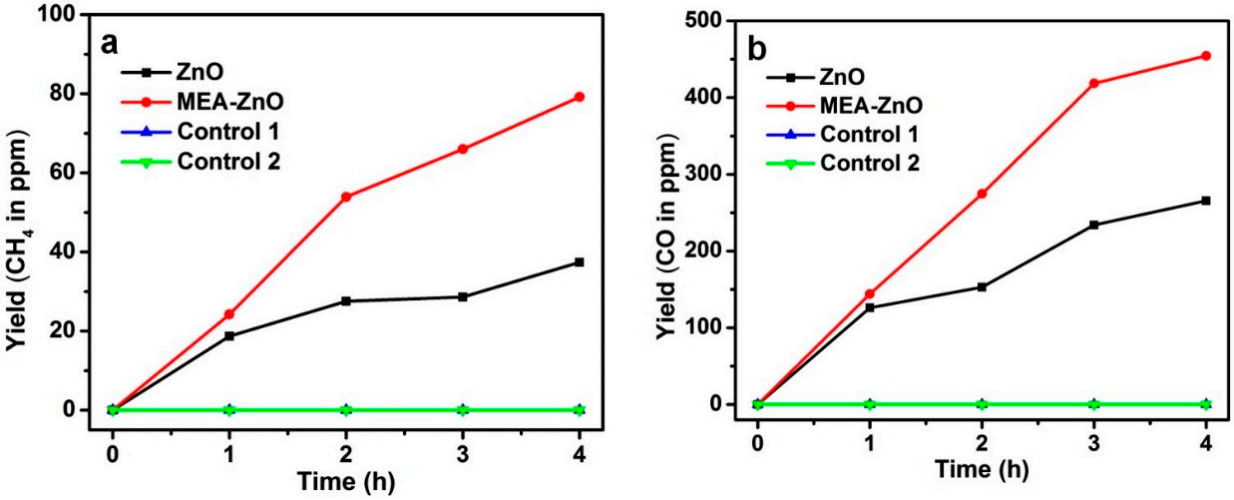
The amount of generated (**a**) CH_4_ and (**b**) CO as a function of irradiation time over the sample of MEA–ZnO or clean ZnO. Control experiments without light irradiation (control 1) or without CO_2_ (control 2) showed no generation of CO and CH_4_.

We further carried out photocatalytic CO_2_ reduction by irradiating the prepared sample (MEA–ZnO or clean ZnO) with UV-Vis light in the presence of CO_2_ and water vapor. As shown in Figure 5, the clean ZnO sample exhibits very low generation rates of methane (2.1 μmol/g) and CO (14.8 μmol/g). In comparison, the amine-functionalized sample (MEA–ZnO) showed a much higher production rate for methane (4.4 μmol/g) and CO (25.3 μmol/g). This comparison indicates that the amine functionalization by MEA can not only enhance the capture of CO_2_ molecules but also facilitates their photoreduction into CO and CH_4_. Control experiments were carried out in the absence of light or CO_2_ (in O_2_ atmosphere), which did not lead to generation of CO or CH_4_. This suggests that excitation of ZnO is required to obtain the products (CO and CH_4_) which come from CO_2_ photoreduction rather than the oxidation of MEA by the photogenerated holes of ZnO.

To measure the amount of O_2_ formed during the photocatalytic reaction, we performed a 12 h photocatalytic reaction for the MEA–ZnO catalyst under the reaction conditions described above. The detected amount of H_2_, O_2_, CO, and CH_4_ were 81 ppm, 1145 ppm, 1520 ppm, and 146 ppm, respectively. According to the stoichiometry of the involved reactions (1) to (4) and the amount of gaseous products (H_2_/CO/CH_4_), the O_2_ evolution amount should be around 1092 ppm, which is slightly lower than the actual detected O_2_ amount (1145 ppm). This might be due to the possible generation of liquid hydrocarbon products (e.g., CH_3_OH, HCOOH) from the reactions (5) to (7), which could not be effectively analyzed by the TCD (thermal conductivity detector) of GC (Gas Chromatography). Nevertheless, this result still can prove that the attached MEA on ZnO surfaces are stable against oxidation by the photogenerated holes from ZnO.

2H_2_O + 4h^+^ → 4H^+^ + O_2_ (1)

2H^+^ + 2e^−^ → H_2_ (2)

CO_2_ + 2H^+^ + 2e^−^ → CO + H_2_O (3)

CO_2_ + 8H^+^ + 8e^−^ → CH_4_ + 2H_2_O (4)

CO_2_ + H^+^ + 2e^−^ → HCOO^−^ + H_2_O (5)

CO_2_ + 4H^+^ + 4e^−^ → HCHO + H_2_O (6)

CO_2_ + 6H^+^ + 6e^−^ → CH_3_OH+ H_2_O (7)

**Figure 6:**
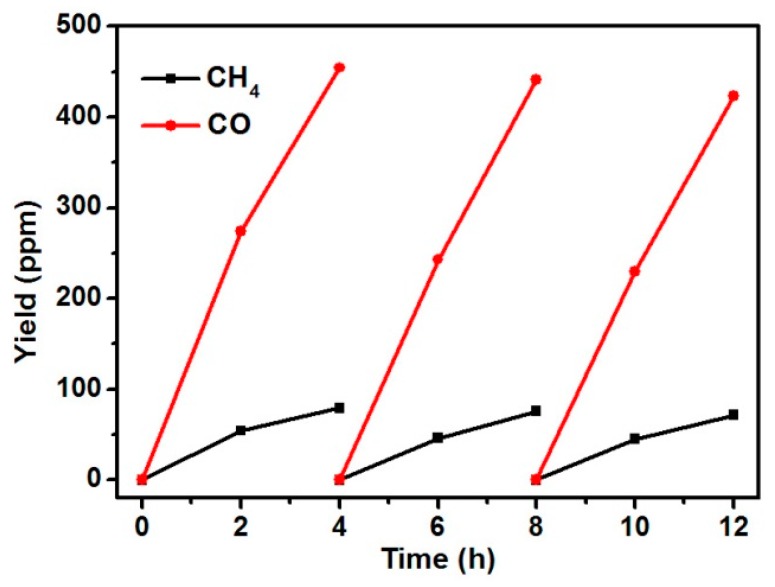
The cycling test of CO_2_ photoreduction over 12 h by using the MEA–ZnO sample.

We have also evaluated the long-term stability of MEA–ZnO for CO_2_ photoreduction through a cycling test. After each cycle of the four-hour test, the reactor was degassed by CO_2_ and the sample was re-used for the next test. As shown in Figure 6, no obvious decrease of the yield of CH_4_ and CO was observed after three cycles, suggesting that the MEA–ZnO sample is very stable during the process of CO_2_ photoreduction. After the cycling test, the MEA–ZnO sample was examined again by FT-IR spectroscopy, and no change was observed in the IR spectrum, which further confirmed the good stability of MEA–ZnO during the reaction.

**Figure 7:**
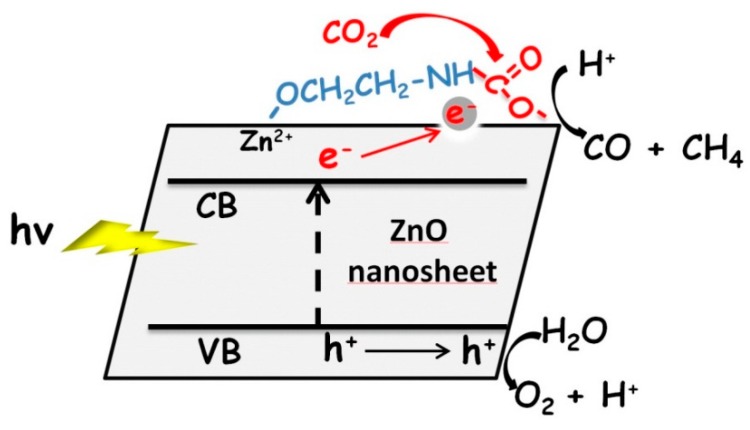
Schematic illustration of adsorption and photoreduction of CO_2_ on the MEA-functionalized ZnO nanosheet.

Based on the above results, we conclude that the amine functionalization of ZnO can effectively facilitate the capture of CO_2_ as well as its further photoconversion into CH_4_ and CO. The principle can be illustrated in Figure 7. The terminal amine groups enable the creation of “C–N” bonding with CO_2_ by forming carbamate, which acts like an activation process of CO_2_ due to higher reactivity of carbamate than that of linear CO_2_ [31,32,33,34]. Further, these active carbamates are close to the ZnO surface and tend to establish direct interactions with Zn^2+^, which allows for receiving electrons from excited ZnO to implement reduction reactions towards CO and CH_4_ production. By this method, the overall CO_2_ photoreduction process can be promoted.

## 3. Experimental Section

### 3.1. Preparation of Amine-Functionalized ZnO (MEA–ZnO)

The amine-functionalized ZnO sample was prepared by a hydrothermal method. In a typical procedure, a mixture aqueous solution (20 mL) containing 0.1 M Zn(Ac)_2_, 0.2 M NaOH, and 0.005 M MEA was put in a Teflon-lined autoclave and heated at 90 °C for 12 h. Then the solid product was collected and annealed at 250 °C under Ar atmosphere for 3 h to remove physisorbed MEA on ZnO surfaces. For comparison, the clean ZnO sample was also prepared as the control sample under the same condition without using MEA.

### 3.2. Characterizations

X-ray powder diffraction (XRD) analysis was carried out on a Shimadzu XRD-6000 X-ray diffractometer (40 KV, 40 mA, Shimadzu Corp., Kyoto, Japan). The sample morphologies were examined by scanning electron microscopy (SEM, JEOL JSM-7600F, JEOL Ltd., Tokyo, Japan) and transmission electron microscopy (TEM, JEOL JEM-2100F, JEOL Ltd.). UV-Vis diffuse reflectance spectra (DRS) were recorded over the spectral range 320–800 nm on a Lambda 750 UV/Vis/NIR spectrophotometer (Perkin Elmer, Waltham, MA, USA). BaSO_4_ was used as a reflectance standard. Fourier transform infrared spectra (FT-IR) were collected on a Frontier FT-IR/NIR spectrometer (Perkin Elmer). The Brunauer–Emmett–Teller (BET) surface areas and CO_2_ adsorption test were measured on a Micro-meritics ASAP 2020M^+^C system (Micromeritics Instrument Corporation, Norcross, GA, USA). 

### 3.3. Photoreduction of CO_2_

The photocatalytic CO_2_ reduction was performed in a 100 mL gastight reactor with a quartz window and two side-sampling ports. In a typical process, the MEA–ZnO sample (20 mg) was suspended in an ethanol solution and the suspension was dispersed on a 6.25 cm^2^ glass slide upon drying. The glass slide containing MEA–ZnO was then put into the gastight reactor. Prior to the irradiation, the reactor was purged with high purity CO_2_ for 30 min to remove the residual air and then 0.1 mL ultrapure water was injected into the reaction system. A xenon lamp (MAX-302, Asahi Spectra Co. Ltd., Tokyo, Japan) was used as the light source for the photocatalytic reaction. During the reaction, the gas product was analyzed periodically through a gas chromatograph (GC-7890A, Agilent Technologies Inc., Santa Clara, CA, USA) with a TCD detector.

## 4. Conclusions

In summary, we have successfully prepared amine-functionalized ZnO nanosheets through a hydrothermal process. The amine functionalization on ZnO surfaces substantially increases the capability of CO_2_ capture via chemisorption with effective CO_2_ activation. As such, amine-functionalized ZnO nanosheets exhibit significantly enhanced photocatalytic activity for CO_2_ reduction into CH_4_ and CO as compared to the clean ZnO.
